# Hydrodynamic Characteristics of Different Undulatory Underwater Swimming Positions Based on Multi-Body Motion Numerical Simulation Method

**DOI:** 10.3390/ijerph182212263

**Published:** 2021-11-22

**Authors:** Jin Yang, Tianzeng Li, Zhiya Chen, Chuan Zuo, Xiaodong Li

**Affiliations:** 1 Institute of Physical Education, Hunan University, Changsha 410082, China; jinyang@hnu.edu.cn (J.Y.); lixiaodong123@hnu.edu.cn (X.L.); 2 School of Industrial Design and Ceramic Art, Foshan University, Foshan 528011, China; zhiya_chen@fosu.edu.cn; 3 School of Sports Science, Shanghai University of Sport, Shanghai 200438, China; 1821519002@sus.edu.cn

**Keywords:** water depth, water surface, computational fluid dynamic, hydrodynamic characteristic, swimming performance

## Abstract

The study of hydrodynamic characteristics of swimming is the main way to optimize the swimming movement. The relationship between position, water depth, and swimming performance of undulatory underwater swimming are one of the main concerns of scholars. Therefore, the aim of this study is to analyze the swimming performance of three different undulatory underwater swimming positions under various swimming depths using a numerical simulation method based on multi-body motion. The simulation was conducted using 3D incompressible Navier–Stokes equations using the RNG *k-ε* turbulence closure equations, and in combination with the VOF method thus that we could include the water surface in our calculations. Different swimming depths based on the distance from the shoulder joint center to the initial water surface were considered. The velocity of the shoulder joint center was captured with a swimming motion monitoring system (KiSwim) and compared with the calculated results. The study found that there was a significant difference in the hydrodynamic characteristics of the three undulatory underwater swimming positions (i.e., the dorsal, lateral, and frontal positions) when swimming near the water surface, and the difference decreased as the swimming depth increased. There was a negative correlation (R(dorsal) = −0.928, R(frontal) = −0.937, R(lateral) = −0.930) between the swimming velocities of the three undulatory underwater swimming positions and the water depth (water depth = 0.2–0.7 m) and that the lateral position had the greatest average velocity. Therefore, it is recommended that swimmers travel at least 0.5 m below the water surface in any undulatory underwater swimming position in order to avoid excessive drag forces. As the swimmer approaches the water surface, the lateral position is worth considering, which has better velocity and hydrodynamic advantage than the other two undulatory underwater swimming positions.

## 1. Introduction

Competitive swimming at the Olympic level is so fierce that success or failure is usually measured in seconds or even in hundredths of a second. Swimming performance is determined by the combined effect of thrust force and drag, which is caused by the interaction between the swimmer and the water. The highly effective undulatory underwater swimming technique can minimize drag force and maximize thrust force [1]. Thus, undulatory underwater swimming (UUS) has been regarded as the “fifth stroke”. It is widely used in conjunction with the butterfly, back, and front crawl strokes as well as after diving or during turning and gliding stages [2]. UUS plays a significant role in overall swimming performance. Much research has analyzed the hydrodynamic characteristics.

Experimental studies have been the main research methods used in the analysis of UUS. Motion diagrams have been used to examine kinematic law and how best to optimize the technique. For example, Jensen et al. [3] estimated the joint reaction forces involved in UUS by modeling the lower extremity of two swimmers at the international level. They found that UUS was more effective for female swimmers as compared to male swimmers. Yamakawa et al. (2017) used a motion capture system to analyze the differences in the hip and knee joint movements of eight collegiate male swimmers employing UUS alone and in conjunction with the butterfly stroke [4]. They found that the hip flexion and extension as well as the knee flexion while performing the UUS-augmented butterfly stroke were markedly different, as compared to UUS alone, while there were no differences in the hip adduction/abduction and internal/external rotation movements.

With the rapid development of computer hardware and software, numerical simulation of complex flow processes is now possible. More and more researchers used numerical simulations for studying swimming. In 1996, Bixler et al. [5] were pioneers in the use of computational fluid dynamics (CFD) in swimming research, and their numerical simulation analysis has gradually become a new focus in the field. Lyttle et al. [6] performed quasi-steady simulations of UUS using CFD. They found that the thrust force came mostly from the legs of a swimmer, as compared to the feet. This conclusion, however, was the result of the computed hydrodynamics and forces on the body that would be significantly different under real-world conditions. Yamakawa et al. (2020) evaluated the effects of the maximum angle of the knee in competitive swimming using the moving computational domain (MCD) method. They found that a larger knee joint angle may improve UUS performance [7]. Mittal et al. [8] performed a high-fidelity simulation analysis of the UUS technique using an immersed boundary method. Using the same approach, Von Loebbecke et al. [9] estimated the propulsive efficiency of swimmers versus cetaceans, and their simulations indicated that the propulsive efficiency of human swimmers varied over a relatively wide range, from about 11% to 29%, which was significantly lower than that of the cetacean. Cohen et al. [2] investigated the effects of changes in ankle flexibility and stroke frequency using the smoothed-particle hydrodynamic method. They found that the net streamwise force on the swimmer was relatively insensitive to ankle flexibility but was strongly dependent on kick frequency. These simulation studies investigating UUS examined the propulsive source, propulsive efficiencies, ankle flexibility, and kick frequency, all of which have made important contributions to swimming research and our understanding of the technical aspects of UUS.

Furthermore, researchers have found that a number of factors contribute to the performance of UUS, including the position, velocity of mass center, kick frequency, kick amplitude, and swimming depth [2]. Previous studies analyzed UUS performances at the same swimming depth. For example, Cohen et al. [2] and Von Loebbecke [9] both built numerical 3D models to study the characteristics of UUS where the water depth was not considered. However, few studies have analyzed the hydrodynamic characteristic of UUS performance at various swimming depths and with regard to the water surface [10,11,12]. In competitive swimming, the frontal position, the lateral position, and the dorsal position are employed at the beginning of a lap and during turns. Swimmers need to choose a suitable UUS position that avoids excessive wave drag, especially as they approach the water surface since wave drag plays a major role in performance. Studies have shown that the contribution of the wave drag to the total drag at the water surface can be 20–50%, depending on the swimming velocity and swimming depth [13,14,15]. This suggests the importance of minimizing the wave drag caused by the interaction between UUS and the water surface. Therefore, studying the hydrodynamic characteristics of different UUS positions at various swimming depths will help swimmers achieve maximum propulsive efficiency at the start of a lap and during turns.

The aim of this study was to analyze the swimming performance of three different UUS positions using a numerical simulation method based on multi-body motion. The deformation movement of the human body is transformed into the rigid body orientation change movement of each independent limb, which avoids the mesh deformity caused by the large change of body in the traditional dynamic mesh numerical method, and can realize the simulation of complex swimming movement. This study also explores the changes in drag and thrust as well as the effect of breaking the surface of the water. It was hypothesized that there was a correlation between different water depths and swimming performance when breaking the water surface.

## 2. Numerical Method

### 2.1. Swimmer Geometry and Motion Capture

**Figure 1:**
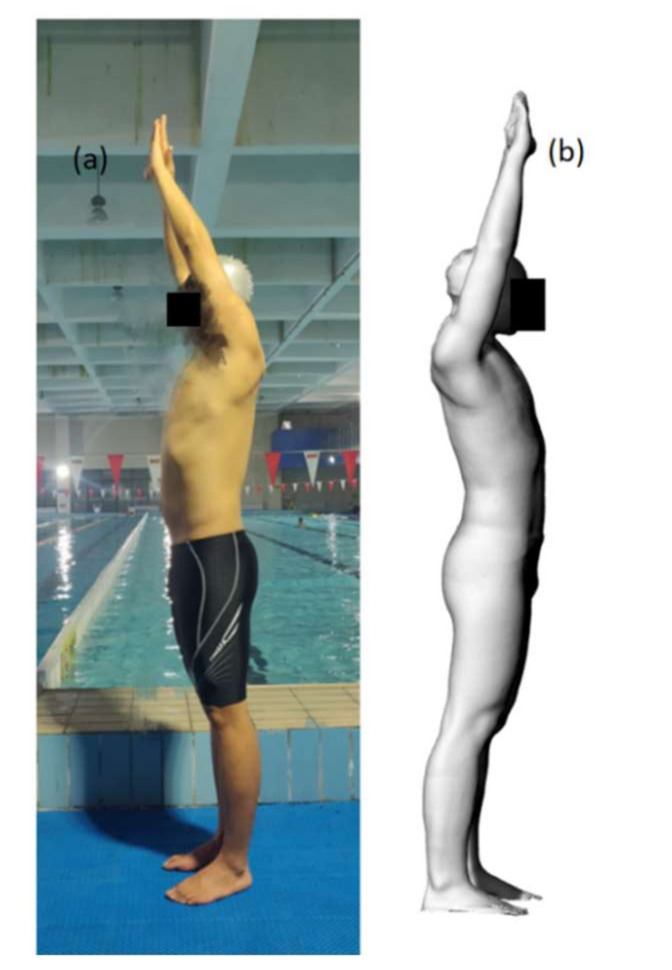
(**a**) Swimmer. (**b**) Surface reconstruction produced from a laser scan.

An elite international swimmer from China volunteered to be the research subject. The ratio of chest, waist, and hip circumferences was 1.17:1:1.16. The mass of the swimmer was 81 kg. First, body shape data from the swimmer’s streamlined standing posture was obtained using a 3D scanning device (ZBOT SCAN-1X). Subsequently, a full-scale swimmer CAD simulation model was created using the reverse reconstruction method (see Figure 1). The participant was fully informed about the experimental protocol and its potential risks and benefits, and written consent was obtained from the participant before testing. The study was conducted in accordance with the Declaration of Helsinki, and the protocol was approved by the Ethics Committee of Physical Education Institute of Hunan University.

The swimmer’s motions were recorded underwater with the swimming motion monitoring system, model KiSwim (including 5 high-speed video cameras). The frame resolution was 1920 × 1080 pixels, and the frame rate was 100 frames/s. This increase in temporal resolution was particularly important in UUS, which, in this case, was 0.48 s, corresponding to 48 frames per swimming cycle. This present stroke period of 0.48 s was similar to the average value of 20 athletes, as measured by Gavilán et al. [16]. 

Human swimming is challenging to simulate using computational fluid dynamics because of the deformations of the body during motion. The multi-body motion numerical simulation method overcomes many of these difficulties. For the numerical swimming simulation, the swimmer’s body was assumed to be either rigid or deformable in many studies. For example, Nakashima [17] proposed the swimming human model (SWUM) simulation, which used independent, rigid, truncated elliptic cones to estimate swimmers’ motions. This method provided practical information for motion optimization and effectively improved the quality of swimming training. 

In our study, the swimmer model was assumed to be a rigid body and ignored factors such as human muscle elasticity and skin texture. The joints of the swimmer model were allocated based on the laws of swimming movements, as defined by [18,19]. The body deformations involved during movement were translated into their rigid-body equivalents for each independent limb, thus as to avoid grid aberrations caused by body deformations. This allowed us to calibrate the movements of the joints without introducing errors. Next, the corresponding joints were each separated, creating uniformly spaced gaps between the rigid body parts and ensuring that they would not collide during movement. The movements of the human body were then replaced by the corresponding rigid body movement, which was limited to displacement and rotational movement, thereby eliminating issues related to shape deformations. Thus, the swimmer’s motions could be replicated by the rigid body model. 

**Figure 2:**
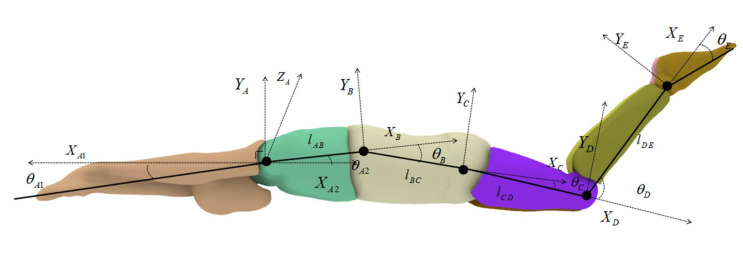
Diagram of the D–H model of Figure 2 is the D–H model schematic diagram of the undulatory underwater swimming. *A*, *B*, *C*, *D*, and *E* represent the shoulders, upper waist, hips, knees, and ankles, respectively, and *l_AB_*, *l_BC_*, *l_CD_*, and *l_DE_* are the distances from *A* to *B*, *B* to *C*, *C* to *D*, and *D* to *E*, respectively. Variables *θ_A_*_1_ and *θ_A_*_2_ are the angles between the limb and the horizontal position; *θ_B_*, *θ_C_*, *θ_D_*, and *θ_E_* are the angles between the adjacent joints.

In this research, the body movements of the swimmer were simplified to 2-dimensional movements in the sagittal plane. The swimmer model was separated into 6 independent rigid bodies, which corresponded to the arms, chest, waist, thighs, shanks, and feet. The motion capture was achieved by constructing motion equations, and the swimmer’s body motion equation was derived from the Denavit–Hartenberg (D–H) parameters [20]. In this paper, the base coordinates of the swimmer model were established by setting the shoulder joint as a reference point. Meanwhile, the posture and the position of the terminal limb relative to the base coordinate system were determined by the continuous multiplication mode of the sub-transformation matrix. Figure 2 shows the D–H model schematic diagram of UUS, and the position transformation matrix between limbs can be described as:

(1) $$\begin{array}{cc} \; & {}_{i+1}^{i}T=Rot\left(z,\theta_{i-1}\right)Trans\left(0,0,d_{i+1}\right)Trans\left(a_{i+1},0,0\right)Rot\left(x,\alpha_{i+1}\right)\\ \; & =\left[\begin{array}{cccc} c\theta_{i+1} & -s\theta_{i+1} & 0 & 0\\ s\theta_{i+1} & c\theta_{i+1} & 0 & 0\\ 0 & 0 & 1 & 0\\ 0 & 0 & 0 & 1 \end{array}\right]\times \left[\begin{array}{cccc} 1 & 0 & 0 & 0\\ 0 & 1 & 0 & 0\\ 0 & 0 & 1 & d_{i+1}\\ 0 & 0 & 0 & 1 \end{array}\right]\\ \; & \times \left[\begin{array}{cccc} 1 & 0 & 0 & a_{i+1}\\ 0 & 1 & 0 & 0\\ 0 & 0 & 1 & 0\\ 0 & 0 & 0 & 1 \end{array}\right]\times \left[\begin{array}{cccc} 1 & 0 & 0 & 0\\ 0 & c\alpha_{i+1} & -s\alpha_{i+1} & 0\\ 0 & s\alpha_{i+1} & c\alpha_{i+1} & 0\\ 0 & 0 & 0 & 1 \end{array}\right]\\ \; & =\left[\begin{array}{cccc} c\theta_{i+1} & -s{\theta_{i+}}_{1}c\alpha_{i+1} & s{\theta_{i+}}_{i}s\alpha_{i+1} & a_{i+1}c\theta_{i+1}\\ s\theta_{i+1} & c\theta_{i+1}c\alpha_{i-1} & -c{\theta_{i+}}_{i}s\alpha_{i+1} & a_{i+1}s\theta_{i+1}\\ 0 & s\alpha_{i+1} & c\alpha_{i+1} & d_{i+1}\\ 0 & 0 & 0 & 1 \end{array}\right] \end{array}$$

where *c* stands for *cos* and *s* stands for *sin*. The *Z*-axis is the movement axis of the joint. The *X*-axis is the common normal along two adjacent *Z* axes. The *Y*-axis is determined by the right-handed rectangular coordinate system formed by *Z*. Additionally, *a* is the length of the limb, which is the minimum distance between two adjacent *Z* axes, while *α* is the twist angle and is the angle between the adjacent *Z* axes; *θ* is the joint variable, which is the angle between the two adjacent *X* axes. The horizontal distance *d* is the distance between the two common normals, which is equal to the distance between the two *X*-axes. Point *A* is the reference origin point; the motion equations of points *B*, *C*, *D*, and *E* are, respectively, deduced as follows:

(2) TB0=(TA0)(TBA)TC0=(TA0)(TBA)(TCB)TD0=(TA0)(TBA)(TCB)(TDC)TE0=(TA0)(TBA)(TCB)(TDC)(TED)

In this study, the UUS in the stable stage was mainly shown as the movement in a horizontal direction, with slight trunk undulation and a rolling motion. In order to reduce the difficulty of the numerical simulation, this study only considered the motion equation of the swimming direction. Moreover, the shoulder joint also fluctuated slightly in the vertical direction, and the position of the center of mass and the shoulder joint center were roughly at the same level in the swimming direction (i.e., the horizontal direction). Thus, the shoulder joint center was used as a reference point for the horizontal velocity of the mass center and the water depth.

The velocity of mass center was calculated according to Newton’s second law. The equation is as follows:

(3) $$v_{t}=v_{t-\Delta t}+\left(F/m\right)\Delta t$$

where *v_t_* is the swimming velocity in swimming direction, *F* is total force, and *m* is the mass of the swimmer.

**Figure 3:**
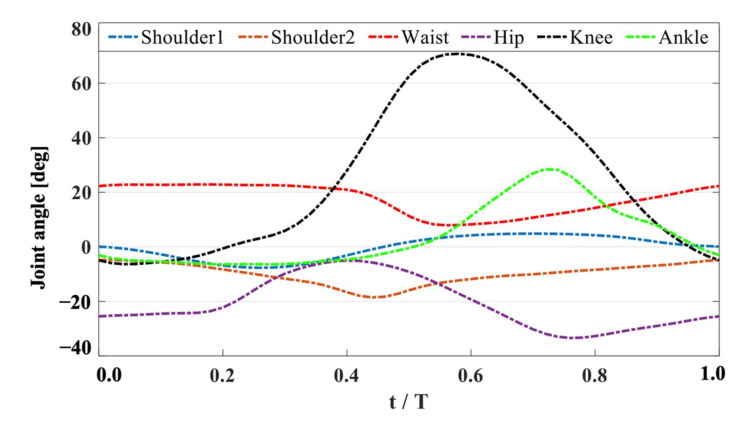
Kinematics of the joints for the UUS of the swimmer.

The joint angle kinematics of the shoulder, waist, hip, knee, and ankle are shown in Figure 3. The joint angle data were collected from video footage analysis of the athlete’s actual movements. The swimming motion monitoring system (KiSwim) assisted in the calibration of each joint position during movement. We obtained the motion angle of each joint based on the joint position. In order to ensure that our trajectory conformed to the real-world conditions, we collected angle data at least three times for the motion trajectory in the video footage. The relationships between these joint angles made up the UUS motion. 

### 2.2. Governing Equations

In this study, the flow was assumed to be an incompressible viscous fluid, described by the continuity equation and the Reynolds-averaged Navier–Stokes equations, including the mass and momentum conservation equations, as follows:

(4) $$\left\{\begin{array}{l} \frac{\partial }{\partial t_{i}}\left(u_{i}\right)=0\\ \frac{\partial }{\partial t_{i}}{\left(u_{i}\right)}_{1}+\frac{\partial }{\partial x_{j}}\left(u_{i}u_{j}\right)=-\frac{1}{\rho }\left(\frac{\partial p}{\partial x_{i}}\right)+g_{i}+\frac{\partial }{\partial x_{j}}\left[v\left(\frac{\partial u_{i}}{\partial x_{j}}+\frac{\partial u_{j}}{\partial x_{i}}\right)-\frac{2}{3}\delta_{ij}\frac{\partial u_{l}}{\partial x_{l}}\right]+\frac{\partial }{\partial x_{j}}\left(-\bar{u_{i}^{\prime }u_{j}^{\prime }}\right) \end{array}\right.$$

where *ρ* is fluid density, *u_i_* and *u_j_* are the components of the velocity vector, *p* is the pressure, *g_i_* is the component of the gravitational acceleration, *v* is the kinematic viscosity, and the index *i*, *j* = 1, 2, 3 for three dimensional flows, $-\bar{u_{i}^{\prime }u_{j}^{\prime }}$ are the Reynolds stresses and must be modeled in a turbulence model. The renormalization group (RNG) *k-ε* turbulence model that has been widely used in many different engineering problems with relatively high precision and reliability was chosen for the closed solution of the system of equations in this study.

(5) $$\left\{\begin{array}{l} \frac{\partial }{\partial t}(\rho k)+\frac{\partial }{\partial x_{i}}(\rho ku_{i})=\frac{\partial }{\partial x_{j}}(\alpha_{k}\mu_{eff}\frac{\partial k}{\partial x_{j}})+G_{k}+G_{b}-\rho \varepsilon +S_{k}\\ \frac{\partial }{\partial t}(\rho \varepsilon )+\frac{\partial }{\partial x_{i}}(\varepsilon u_{i})=\frac{\partial }{\partial x_{j}}(\alpha_{\varepsilon }\mu_{eff}\frac{\partial \varepsilon }{\partial x_{j}})+G_{1\varepsilon }\frac{\varepsilon }{k}(G_{k}+C_{3\varepsilon }G_{b})-C_{2\varepsilon }\rho \frac{\varepsilon^{2}}{k}-R_{\varepsilon }+S_{\varepsilon } \end{array}\right.$$

where *k* is the turbulent kinetic energy, *ε* is the turbulent dissipation rate, *G_k_* is the generation of turbulent kinetic energy due to the mean velocity gradients, *G_b_* is the turbulent kinetic energy that is determined by buoyancy, *μ_eff_* is the turbulent viscosity, *μ_eff_ = μ + μ_t_*, *μ* is the viscosity of water, *μ_t_ = ρC_μ_k*^2^*/ε*, and *R_ε_* is the extra term that expresses the effect of the average strain rate on *ε*. The quantities *α_k_* and *α_ε_* are the inverse effective Prandtl numbers for *k* and *ε,* and *S_k_* and *S_ε_* are user-defined source terms. Additionally, *C*_1*ε*_, *C*_2*ε*_, *C*_3*ε*_, *C_μ_*, *α_k_*, and *α_ε_* are empirical constants.

In order to consider the effect of the water surface during swimming, a two-phase flow, including air and water, was involved in this study. The volume of fluid (VOF) method was used to track the deformation of the air–water interface [21]. Therefore, a new transport equation for the volume fraction of fluid was introduced. *F_q_* denotes the volume fraction and is determined by the following equations:

(6) $$\left\{\begin{array}{l} \frac{\partial F_{q}}{\partial t}+\frac{\partial }{\partial x_{i}}(F_{q}u_{i})=0(q=1,2)\\ F_{1}+F_{2}=1 \end{array}\right.$$

where *F_q_* = 1, when the cell was filled with the fluid of the *q*-th phase; in turn, for *F_q_* = 0, when the cell did not contain any fluid of the *q*-th phase. Additionally, for 0 < *F_q_* < 1, the cell was called an interface cell; *q* = 1 or 2, since there were only two phases, namely air and water. Additionally, the swimming movement was carried out by applying a dynamic mesh method in which the meshes of the domain were adjusted according to the timing and the motions of the swimmer. The integral form of the conservation equation for a general scalar *ϕ*, on an arbitrary control volume *V*, whose boundary is moving, can be written as:

(7) $$\frac{d}{dt}\int_{V}\rho \phi dV+\int_{\partial V}\rho \phi (\vec{u}-{\vec{u}}_{g})\cdot d\vec{A}=\int_{\partial V}\Gamma \nabla \phi \cdot d\vec{A}+\int_{\partial V}S_{\phi }dV$$

where $\vec{u}$ is the flow velocity vector, ${\vec{u}}_{g}$ is the mesh velocity of the moving mesh, *Γ* is the diffusion coefficient, and *S_ϕ_* is the source term of *ϕ*.

### 2.3. Computational Domain

**Figure 4:**
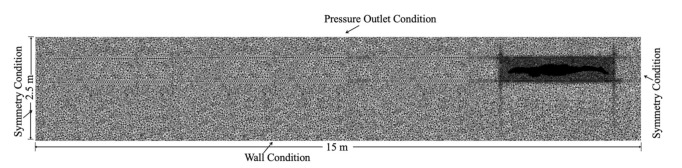
Computational domain.

A numerical 3D flume with the dimensions of 15 m × 2.0 m × 2.5 m was used. The water depth was 2 m, and the air height was 0.5 m. The distance between the reference point (shoulder joint center) and the initial water surface was defined as the swimming depth. The swimmer model was placed in the mid-line of the flume. The detailed boundary settings were described in our previous research [22]. The computational domain was set as the active zone, which was a deformable mesh zone where the mesh updated with time and was made up of tetrahedral types (see Figure 4). Based on the characteristic length of 0.2 m of the swimmer’s transverse section, the Reynolds number was approximately 3.6 × 10^6^ in order to obtain more reliable calculation results, which was based on our previous findings [22]. In this study, the grid near the swimmer’s surface was set as 0.005 m, and the overall y+ was between 32 and 160.

### 2.4. Numerical Implementation

The ANSYS FLUENT V19.0 was used for the fluid simulation. The RNG *k*-*ε* turbulence model was used. The finite-volume method was used to determine the governing equations, coupled with the pressure velocity by the pressure-implicit with the splitting of operators (PISO) method, and the pressure interpolation was set as the second order. In addition, the second-order upwind was used for separate convection terms, while the first-order implicit was used for the time change term. Additionally, spring-based smoothing and local-cell-remeshing methods were used to update the mesh. The motion capture was realized by a secondary development of the User Defined Function (UDF).

### 2.5. Statistical Analysis

The statistical analysis was performed using SPSS Statistics 19.0. Associations between the variables of USS (including lateral, frontal, and dorsal positions) and water depth were assessed using bivariate correlations. Assessment of Pearson correlation coefficient was performed to evaluate the main effect of the water depth on the swimming velocity of the three UUS positions. Results were considered significant at *p* < 0.05.

## 3. Results and Discussion

### 3.1. Validation of the Velocity

**Figure 5:**
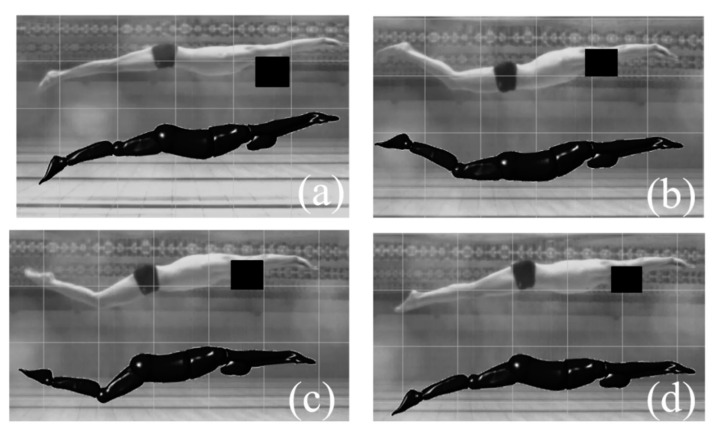
The frontal position of the swimmer alongside the corresponding frame of the video footage. (**a**,**b**) corresponds to the flexion kick of the UUS; (**c**,**d**) corresponds to the extension kick of the UUS.

The surface of the swimmer’s body was carefully animated in order to visually match each frame accurately. This was performed by manipulating the joints of the swimmer model alongside the video footage using the UDF code within ANSYS software, as shown in Figure 5. In addition, the swimming action and velocity of the participant in the stable stage were extracted. A UUS cycle was about 0.48 s, the average water depth at the shoulder center point was 0.45 m, and the average velocity of the shoulder center point of the subject was 1.8 m·s^−1^). The data from the video footage data were numerically simulated as trajectory and velocity boundary conditions for the model.

**Figure 6:**
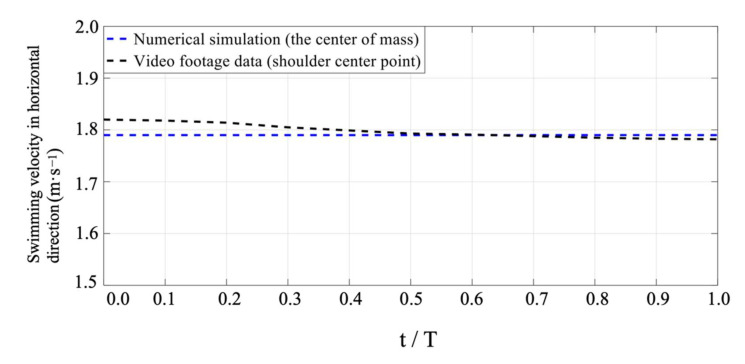
Comparison of video and numerical results in horizontal direction.

In comparison to the velocity of the shoulder center point at the same water depth, the velocity of mass center under the current numerical simulation was very close (see Figure 6). The video footage captured the velocity of the shoulder center point. The shoulder center fluctuated with the rotation of the arm during swimming. This indicated that there were only subtle velocity surges that occurred throughout the stroke while below the surface, which was consistent with the findings of Connaboy et al. (2009) and Colman et al. (1999). Colman et al. [23,24] investigated the velocity variation of a swimmer’s center of mass using the water visualization method. They found that the velocity of mass center during the stroke was constant. Additionally, the velocity of mass center, as a velocity of simulation, was also commonly used in tethered simulations [25,26]. We also found that this velocity of mass center was realistic, as was shown in several of our recent video footage analyses of UUS. Therefore, the subject’s velocity of mass center was used to simulate the swimming velocity in this study.

### 3.2. Net Streamwise Forces

**Figure 7:**
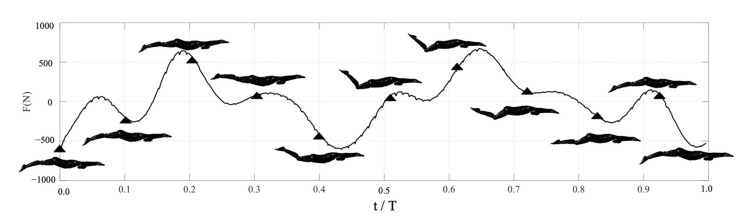
Corresponding diagram of instantaneous action and net streamwise forces.

The net streamwise forces of the swimmer on the sagittal plane throughout one stroke cycle were presented, as shown in Figure 7. The positive force corresponded to a net thrust, and a negative force corresponded to a net drag. The large increase in streamwise force and the high peak occurred during thrust when the legs were at the mid-point of flexion (t ≈ 0.0–0.2 T). This corresponded to the minimums in the frontal areas. The large decrease in streamwise force occurred during stroke reversal when the knee had reached maximum flexion (t ≈ 0.2–0.45 T). This corresponded to the maximum in the front area of the swimmer, which was the cause of the increase in shape drag. There was a short peak in thrust prior to the extension kick (t ≈ 0.45–0.82 T). Finally, there was a short, lower peak in thrust at the end of the extension kick (t ≈ 0.82–1.0 T).

The UUS action consists of two phases: flexion kick, with the feet and legs moving towards the surface of the water, and the extension kick, with the feet and legs moving towards the bottom of the swimming pool [27]. This produces a lot of thrust in both the flexion kick and extension kick, which was consistent with the findings by Atkison et al. on UUS performance [28]. They found that the extension and the flexion kick phases were related to UUS performance. Using an underwater video camera, they studied 26 competitive swimmers, ranging in level from provincial to international. They found that the flexion kick phase performance had the greatest impact on UUS performance. This was significant because qualitative observations had suggested that the extension kick produced most of the thrust for the swimmer. Our video footage analysis, combined with numerical simulations, showed that the thrust force generated from the end of the extension kick to when the legs reach the mid-point of flexion was also significant. It can be seen that increasing the knee flexibility and muscle strength during the flexion kick phase will improve the propulsion efficiency, which is similar to the reporting of Willems et al. [29] for ankle flexibility and muscle strength. They investigated the effect of ankle flexibility and muscle strength in UUS performance by conducting trials with 26 competitive swimmers. They found that UUS velocity may be enhanced via ankle strengthening exercises and that subjects with restricted ankle flexibility may benefit from a flexibility program. Thus, for an athlete, they may need to focus on optimizing the flexibility and amplitude of their flexion kick while working to strengthen the required musculature.

**Figure 8:**
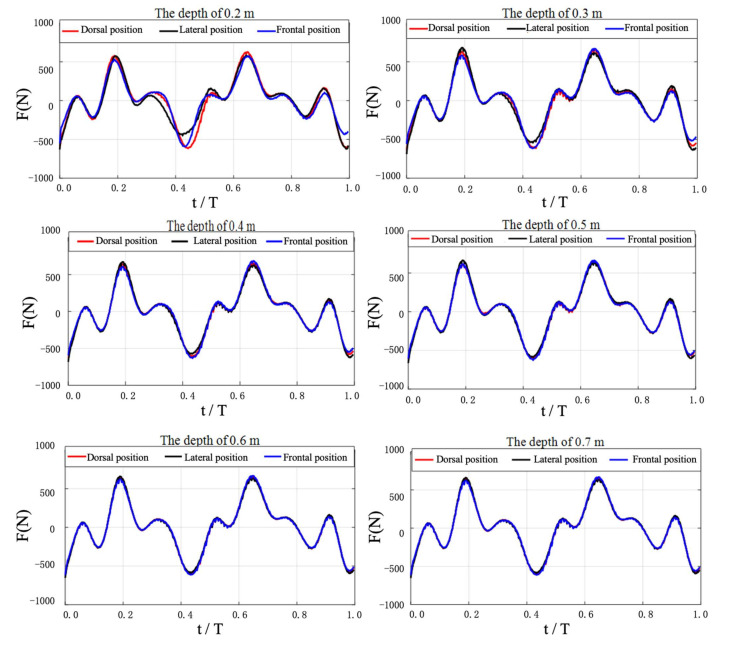
Evolution correspondence between water depth and net streamwise force.

Figure 8 shows the net streamwise forces evolution of three UUS positions at different water depths. The different water depth curves had similar force profiles with no phase differences when the time was non-dimensionalized by the kicking period, T. One noticeable difference was that shallower depth instances have lower peaks and troughs in the net streamwise forces. The net streamwise forces, in contrast, were basically the same when the swimming depth was lower than 0.5 m. As the swimming depth decreased, the net streamwise forces corresponding to the three UUS positions began to show differences in the range of 0.25–0.55 T. The net thrust produced was roughly similar, but the lateral position had the least net drag. In fact, the above phenomenon was similar to the generation of wave drag near the water surface. When the swimmer moved closer to the water surface, the surface was disturbed and deformed, resulting in wave drag. For UUS near the water surface, the wave drag caused by the violent interaction between the limbs and the water surface offset the propulsive force generated by kicking, resulting in a decrease in propulsion efficiency. Therefore, adjusting the stroke technique in order to suppress wave generation may improve the performance of the butterfly kick. In addition, the calculated results showed that the hydrodynamic performance of the three UUS positions was basically the same when the water depth was lower than 0.5 m. This suggested that the effect of wave drag had decreased to the minimum value, which would be desirable for efficient UUS at the start of a lap and when turning.

In the previous hydrodynamic studies, the results for different UUS positions have been inconsistent. For example, Arellano et al. studied the influence of variance between the three body positions and found significant hydrodynamic differences [30]. However, Von Loebbecke et al. prerecorded video footage of 9 female and 13 male Olympic-level athletes swimming underwater in order to analyze their strokes. They found that while some swimmers performed the UUS in a dorsal position, others did so in a frontal or lateral position, and no distinctions were made between these positions when considering the results [31]. Current swimming researchers have suggested that the reason for these two different conclusions may be that the athletes in the numerical simulation studies were able to maintain similar UUS techniques in different positions. In the actual swimming test, these kinematic differences were attributed to the swimmers’ lack of familiarity with the different positions.

### 3.3. The Swimming Velocity

**Figure 9:**
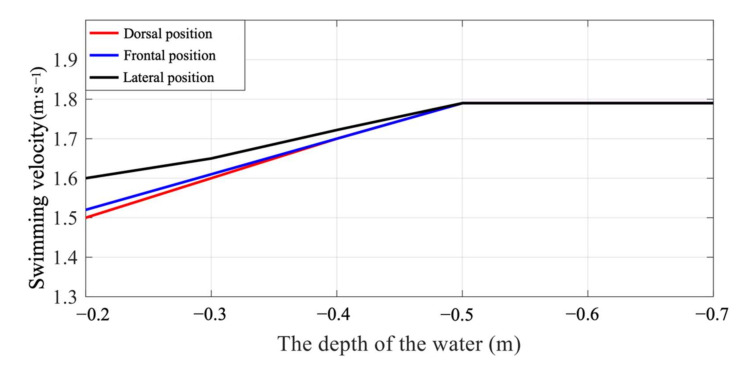
The swimming velocity of the three swimming positions at different water depths.

The swimming velocities of the frontal, dorsal, and lateral positions were calculated at a water depth from 0.2 m to 0.7 m, as shown in Figure 9. The lateral position had the maximum velocity, followed by the frontal and dorsal positions, which increased proportionally to water depth. The velocities of the three UUS positions were similar at a water depth of 0.5 m, and the swimming velocity was not sensitive to the increase in water depth. This was largely due to the significant reduction in wave drag as the swimmer traveled at greater depths, which was consistent with the findings by Elaine et al. on wave drag [32]. They measured the total drag force, as well as the specific contribution of wave drag during the underwater phase at the beginning of a lap, using the towed method. They found large decreases in total drag as the depth increased (e.g., 19.7% at 0.5 m and 23.8% at 1.0 m). The towing method, while appropriate for their research, generated a gliding phase test in, essentially, an ideal state. In contrast, our focus was to simulate complex swimming dynamics that were not subject to the influence of the swimmer, which would help to ensure that our results remained objective. It has been recommended that swimmers travel at least 0.5 m below the surface in order to avoid excessive drag. As the swimmer approaches the water surface, the swimmer may use the lateral position, which has more swimming velocity and hydrodynamic advantages than the other two UUS positions.

Table 1 presents the comparisons between the swimming velocity of the three UUS positions and water depth. These variables were negatively correlated. The selected model was statistically significant. The swimming velocity of different UUS positions increased proportionally with water depth. In addition, compared to the dorsal and frontal positions, the lateral position has the highest average velocity. This indicated that the lateral position in competitive swimming might be more efficient, which was also consistent with the net streamwise force of the three UUS positions shown in Figure 8.

### 3.4. Visualization Analysis

#### 3.4.1. Surface Pressure

**Figure 10:**
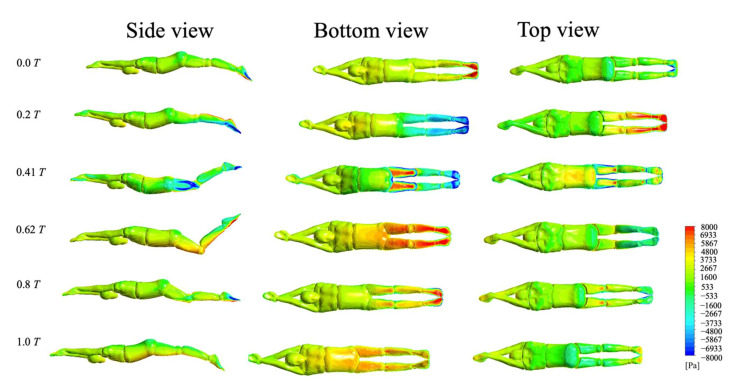
Evolution diagram for the surface pressure of athletes.

Figure 10 shows the surface pressure changes of the swimmer’s body during frontal UUS. As can be seen from the figure, the frontal kick consisted of two stages: the flexion kick (often referred to as the “up-kick”) and the extension kick (often referred to as the “down-kick”). In the process of the flexion kick, the hip joint moved counterclockwise, and the lower limbs pushed the water to flow obliquely behind. A high-pressure area was formed at the bottom of the foot (t ≈ 0.2 T), resulting in an increase in propulsion force. In the process of the extension kick, the front of the leg and the back of the foot also formed a high-pressure area due to the rapid push through the water and surge behind the syncline (t ≈ 0.62 T), which resulted in a rapid increase in thrust force. Additionally, the angle of attack between the shank and the horizontal direction gradually increased during the stroke reversal when the knee had attained maximum flexion (t ≈ 0.2–0.41 T). The shank and the instep had become the areas of action that blocked the flow of the water, forming a low-pressure area. The swimmer’s body was surrounded by large pressure differences, which led to a sharp increase in the athlete’s overall resistance. In general, the upward and downward kick of the lower limbs directly affected the pressure distribution surrounding the swimmer, leading to changes in the pressure between the front and the back of the swimmer.

#### 3.4.2. Velocity Vector of Flow Field

**Figure 11:**
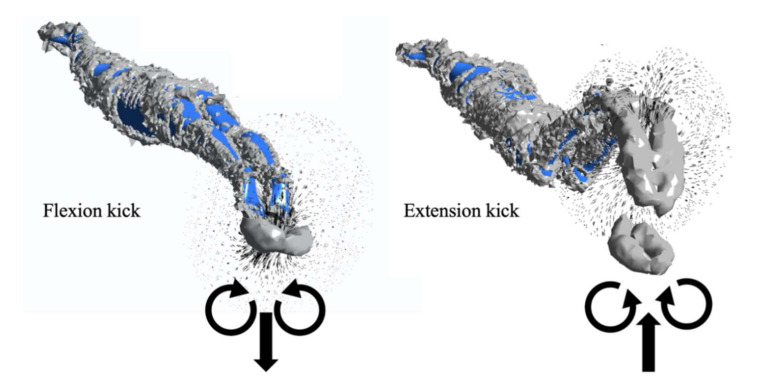
Two counter-rotating vortices and a jet stream.

Figure 11 shows the velocity vector of the flow field and the rotation direction of the vortices generated after each change in the kick movement. The thrusting impulse was a reaction to the jet stream away from the body, moving between the counter-rotating vortices [33]. When the swimmer completed a flexion kick, the flows at the cross-section of the toe produced a strong downward jet stream. Two counter-rotating vortices were generated in the wake of the swimmer’s feet, and the water flows converged at the place where the feet had been together. When the swimmer completed an extension kick, the water flowed up to the cross-section of the toe and produced a strong upward jet stream. It is precisely because of this that the flexion kick and extension kick can produce strong propulsion (see Figure 9).

The net streamwise force was deeply involved with the vortex rings generated by the swimmer’s motions. In fact, various highly adapted aquatic organisms also produce vortex wakes when they swim, and the shape and arrangement of the wakes are directly related to swimming velocity [34]. Videler et al. used particle imagery velocimetry to study fish swimming [35]. They found that the wakes produced by the fish consisted of two vortices per tail-beat cycle. A jet of water undulated between the vortices and flowed opposite to the swimming direction, which was also similar to our findings.

#### 3.4.3. Water Surface

**Figure 12:**
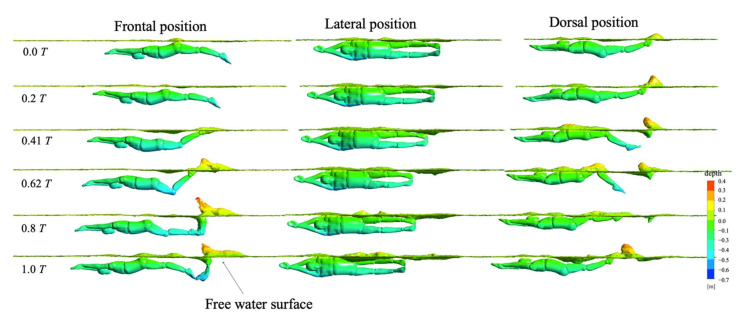
Wave evolution diagram near the water surface.

Figure 12 shows the transformation of the water surface corresponding to the three UUS positions at a depth of 0.2 m. The vertical wave deformation of the water surface during frontal and dorsal positions was more significant than that of the lateral position. The reason for this phenomenon was that the frontal and dorsal kicking directly affected the rise and fall of the water surface, forming a larger wave. The influence of a frontal or dorsal position was an interesting aspect due to many athletes adopting one or the other during competitions. The dorsal position produced a large wave peak at 0.0 T while the frontal position only produced a wave peak at 0.62 T. Correspondingly, we could infer that the wave drag generated by the dorsal position would be the earliest, which was consistent with the findings of Pease et al. on wave drag in human swimming [36]. Their research showed that the dorsal orientation exhibited an earlier onset of wave drag than the frontal position. This was likely due to the flow along the ventral surface of the body being more disturbed than on the dorsal, and thus, the flow field interacted with the surface earlier, creating more substantial waves [37]. The wave peak produced by the lateral position was much lower than that of the other two UUS positions at a depth of 0.2 m. This indicated that the net drag from the frontal and dorsal positions was greater, which was also consistent with the difference in the net streamwise forces (see Figure 8). Additionally, the lateral position swings up and down in the sagittal plane. This is similar to the cyclical left–right swing of a fish’s body in the coronal plane. Therefore, the water surface deformation was not a consistent undulating wave; instead, it was a complex disturbance state.

**Figure 13:**
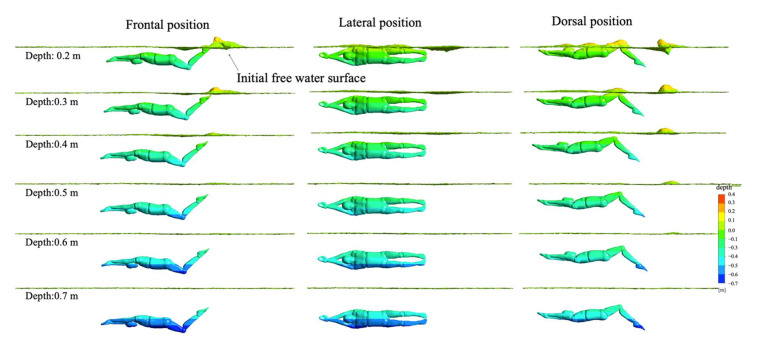
Transformation of water surface under different water depths.

In order to further explore the corresponding relationship between water surface deformation and water depth changes in the three UUS positions, we selected the surface conditions of 0.2 m~0.7 m at 0.62 T in order to complete visual analysis. Figure 13 shows our results. With an increase in water depth, the degree of deformation of the water surface corresponding to the three UUS positions gradually weakened. When the water depth increased to 0.5 m, the water surface remained calm. As inertial waves are typically generated when an object is moving closer to the surface, one of the most significant influencing factors to wave drag is depth [38]. This was also consistent with our research of net streamwise force and swimming velocity (see Figure 8 and Figure 9). In combination with net streamwise force, velocity and water surface were analyzed. As the water depth increased, the swimming velocity increased, and the deformation of the water surface decreased in all three UUS positions. Among them, the lateral position had the least impact on the water surface, followed by the frontal and dorsal positions. Therefore, we inferred that the wave drag gradually decreased as the water depth increased. When near the water surface, the lateral position had the least wave drag and would have the highest swimming velocity, followed by the frontal and dorsal positions. On the contrary, when the water depth was greater than 0.5 m, the wave-making resistance decreased rapidly. The water surface remained calm, and the swimming velocities of the three UUS positions were nearly the same.

In fact, previous research findings were consistent with our current research results. For example, Elaine et al. [32] investigated total drag force as well as the specific contribution of wave drag during the underwater phase using mixed modeling and plotting methods. They found that swimmers had a hydrodynamic advantage when underwater, as compared to swimming on the water surface, with considerably less wave drag when the swimmer remained at least 0.5–0.75 m below the water surface, with no additional benefit below 0.75 m [39]. Our previous study [23] also verified that the wave drag was negligible when gliding depth exceeded body thickness by three times. Thus, we suggest that swimmers travel at least 0.5 m below the water surface to avoid excessive drag forces, which also should improve the efficiency of their underwater kick.

#### 3.4.4. Limitations and Prospects

The UUS consists of simultaneous, vertically oriented motions of the feet. Displacements of the feet and body are normal to the coronal or frontal plane and have minimum magnitude at the shoulders [17]. In this study, it was assumed that the three UUS positions were moving in the swimming direction (horizontal direction). However, ignoring the fluctuation and rotation caused a deviation between the calculated results and the actual situation. Future research should attempt to capture six-degrees-of-freedom motion around the swimmer.

## 4. Conclusions

This study examined UUS as the research object. A rigid-body simulation strategy for UUS was established, which simulated the complex dynamics of UUS swimming. With regard to the impact of water depth on UUS, the influence of different positions as well as the effect of the water surface on the hydrodynamic characteristics of UUS was discussed. The main conclusions are as follows:

(1) The validity of the numerical swimming velocity was demonstrated by comparing the computed results with the experimental data. In addition, this study provides a new method to analyze the hydrodynamic characteristics (drag, thrust, flow field visualization, etc.) of dynamic swimming movement, which is valuable for diagnosing swimming techniques and helps coaches to guide athletes to improve their performance.
(2) There was a significant difference in the hydrodynamic characteristics of the three UUS positions when swimming near the water surface, and the difference decreased as the swimming depth increased. It is, therefore, recommended that swimmers travel at least 0.5 m below the surface in any UUS position in order to avoid excessive drag forces.
(3) As the swimmer approaches the water surface, the swimmer should use the lateral position, which has more swimming velocity and hydrodynamic advantage than the other two UUS positions.
(4) The numerical results showed that the flexion kick could also generate significant propulsion as well as the extension kick. Therefore, competitive swimmers should focus on optimizing the flexibility and amplitude of their flexion kick while working to strengthen the required musculature.

## Figures and Tables

**Table 1:** Relationship between the swimming velocity of the three UUS positions and water depth.

	Water Depth (0.2–0.7 m)		
	R	*p*-Value	Mean (m·s^−1^)
Dorsal position	−0.928	<0.001 **	1.69
Frontal position	−0.937	<0.001 **	1.70
Lateral position	−0.930	<0.001 **	1.72

R: Pearson correlation coefficient. ** Statistically significant with *p* < 0.05.

## Data Availability

The data used to support the findings of this study are available from the corresponding author upon request.
